# Overcoming barriers in myalgic encephalomyelitis/chronic fatigue syndrome research: the CureME participatory model

**DOI:** 10.3389/fnhum.2026.1826525

**Published:** 2026-07-07

**Authors:** Ella Abken, Sarah Tyson, Caroline Kingdon

**Affiliations:** 1 Department of Clinical Research, Faculty of Infectious and Tropical Diseases, London School of Hygiene and Tropical Medicine, London, United Kingdom; 2 School of Health Sciences, Faculty of Biology, Medicine and Health, University of Manchester, Manchester, United Kingdom

**Keywords:** community-engaged recruitment, co-produced study design, ME/CFS, myalgic encephalomyelitis/chronic fatigue syndrome, participatory research

## Abstract

Research into Myalgic Encephalomyelitis/Chronic Fatigue Syndrome (ME/CFS) presents unique challenges. These stem from the logistical difficulties created by the degree of disability experienced and heterogeneous diagnostic criteria driven by etiological uncertainty. This is compounded by distrust of research institutions within the ME/CFS community, due to historical mischaracterization of ME/CFS as a psychosomatic disease. This commentary proposes a framework to address the resulting methodological and practical barriers. The CureME Framework draws on the UK ME/CFS Biobank’s extensive experience of participatory research and incorporates strategies for effective recruitment and data collection. It is informed by collaborations with individuals with lived experience of ME/CFS including severely affected individuals. This is achieved by integrating co-produced study design, community-engaged recruitment, and adaptations that minimize the cognitive and physical burden of participation. This increases access to research participation, reduces selection bias, and strengthens cohort representativeness. Adopting this approach may enhance methodological rigor, accessibility, trust, cohort representativeness, statistical power, and ethical integrity in ME/CFS research.

## 1 Introduction

Myalgic Encephalomyelitis/Chronic Fatigue Syndrome (ME/CFS) is defined as a complex, debilitating multisystem disease with symptoms that persist for at least 3 months and significantly impair daily functioning (Carruthers et al., 2011). It has been classified as a neurological disease by the World Health Organization for over a decade (World Health Organization, 2016), although the cause and mechanism are unclear. However, recent genetic studies have indicated a neuroimmune etiology (Devereux-Cooke et al., 2022) and demonstrating central physiological abnormalities in ME/CFS explained by pathological disease processes have debunked the previous widely held psychosomatic attribution (Thoma et al., 2024).

Consistent with its multisystem nature, ME/CFS is characterized by persistent debilitating fatigue unrelieved by rest, post exertional malaise (PEM), sleep disturbances, cognitive dysfunction, pain, and autonomic symptoms (Committee on the Diagnostic Criteria for Myalgic Encephalomyelitis/Chronic Fatigue Syndrome and Board on the Health of Select Populations, 2015; Fukuda et al., 1994; Samms and Ponting, 2025a). Among these, PEM is the key symptom that distinguishes ME/CFS from other conditions (Jason et al., 2015; Carruthers et al., 2011), defined as the worsening of symptoms following activity. The symptoms can be delayed, and the effect long-lasting (Hartle et al., 2021; Stussman et al., 2020; Chu et al., 2018; Muirhead et al., 2024).

However, despite the severity and breadth of these symptoms, ME/CFS remains difficult to diagnose, with no validated biomarker and reported diagnostic delays of several years. Resulting in imprecise prevalence estimates, with approximately 400,000 people reported in the UK to have ME/CFS (Samms and Ponting, 2025a). ME/CFS affects all ages, ethnicities, and socioeconomic groups, and is most prevalent in middle age, with women diagnosed three to four times more often than men (Samms and Ponting, 2025b; Nacul et al., 2011; Bayliss et al., 2014; Pheby et al., 2020). Most face significant functional limitations with approximately 25% being house- or bed-bound, and up to 75% with limited work capacity (Committee on the Diagnostic Criteria for Myalgic Encephalomyelitis/Chronic Fatigue Syndrome, Board on the Health of Select Populations, Institute of Medicine, 2015). Consequently, people with ME/CFS experience impaired health-related quality of life, with a mean EQ-5D-3 L index of 0.47, reflecting severe functional limitations even after adjusting for comorbidities (Hvidberg et al., 2015; Nacul et al., 2011; Kingdon et al., 2018).

In this commentary we highlight key methodological and practical barriers in ME/CFS research and propose the ‘CureME Framework’ as a way to address them. This was developed by the CureME team at the London School of Hygiene and Tropical Medicine, based on 15 years of research in ME/CFS. Strategies include co-produced study design, community-engaged recruitment, inclusive data collection approaches, and home-based data collection with an emphasis on the needs of severely affected individuals.

We have identified three main barriers to ME/CFS research: identifying and recruiting participants, distrust of research by people with ME/CFS, and practical issues.

## 2 Identifying people with ME/CFS for research studies

As noted above, the cause of ME/CFS is unclear, and therefore no validated diagnostic biomarkers are available. This means diagnosis of ME/CFS, and identification of potential participants rely on symptom-based case definitions (Committee on the Diagnostic Criteria for Myalgic Encephalomyelitis/Chronic Fatigue Syndrome, Board on the Health of Select Populations, Institute of Medicine, 2015; Conroy et al., 2022). This has led to considerable heterogeneity. Over twenty diagnostic criteria exist for ME/CFS, based on expert consensus rather than empirical data (Conroy et al., 2022; Brurberg et al., 2014).

Consequently, there is an urgent need for research to develop validated diagnostic biomarkers and accurate phenotyping methods. In the interim, researchers should apply symptom-based diagnostic criteria clearly and consistently (National Guideline Centre (UK), 2020; Lim and Son, 2020). Achieving this requires transparent protocols, comprehensive documentation, and a willingness to adopt innovative approaches (Huang et al., 2024; Nacul et al., 2020). Multi-stakeholder engagement involving people with ME/CFS, caregivers, clinicians, and researchers would also support the development of consensus-based guidance to standardize and improve recruitment methods (Esmail et al., 2015; Goodman et al., 2020).

However, there is no consensus about which diagnostic criteria should be used in either research or clinical practice. This has led to inconsistent application which undermines the validity and reproducibility of research cohorts while simultaneously excluding individuals from under-represented groups may contribute to sample variation and inconsistent research findings (Fukuda et al., 1994; Arron et al., 2024; Nacul et al., 2018; National Guideline Centre (UK), 2020; Jones and Younger, 2025; Wallerstein and Duran, 2006; Lacerda et al., 2018; Jason et al., 2008; Hunt et al., 2022). Overcoming these limitations requires inclusive, innovative recruitment strategies so that studies capture the full clinical spectrum of ME/CFS (Lacerda et al., 2018; Beentjes et al., 2024; Nacul et al., 2017).

Therefore, the CureME Framework recommends that potential participants must have a clinical diagnosis of ME/CFS and meet Canadian Consensus Criteria (CCC) and/or CDC-1994/Fukuda criteria. as recommended by the European ME Network (EUROMENE) consensus statement (Pheby et al., 2020). Whichever criteria are used, researchers need to explicitly report the criteria they have adopted and precisely define all inclusion and exclusion criteria (Committee on the Diagnostic Criteria for Myalgic Encephalomyelitis/Chronic Fatigue Syndrome, Board on the Health of Select Populations, Institute of Medicine, 2015). When recruiting through clinical services, it should be recognized that clinical diagnostic practices may not align with research case definitions (Jason et al., 2015; Conroy et al., 2022). Any discrepancies need to be identified and resolved during study planning. This will ensure appropriate participant selection and sample validity and reproducibility (Jason et al., 2015; Conroy et al., 2022).

Further, the CureME Framework advises using community-based recruitment, in addition to clinic-based approaches. This is because patients attending ME/CFS services tend to be relatively newly diagnosed and mild-moderately affected (British Association of Clinicians in ME (BACME), 2023). Thus, including community-based recruitment strategies such as ME/CFS patient organizations/charities, support groups and networks can enhance representativeness and reduce selection bias (Kingdon et al., 2020; Pendergrast et al., 2016; Wiborg et al., 2010). However, it can tend to disproportionately recruit individuals with health literacy and/or digital access (O’Boyle et al., 2022; Baker and Hann, 2001; Lacerda et al., 2019). This can be mitigated, to some extent by adopting the full range of digital and non-digital communication strategies used by participating organizations.

### 2.1 Distrust among people with ME/CFS

ME/CFS has a controversial history, having been incorrectly classified as a psychosomatic illness, which contributed to patient stigma (O’Boyle et al., 2022; Wilshire and Kindlon, 2019; Kindlon, 2011). Consequently, symptoms and disabilities were frequently overlooked or misattributed to psychological causes and patients’ testimonies were dismissed (Froehlich et al., 2021). This may be associated with reduced trust in researchers and clinical services, and lower participation in research studies (O’Boyle et al., 2022; Wilshire and Kindlon, 2019; Kindlon, 2011).

**Figure 1:**
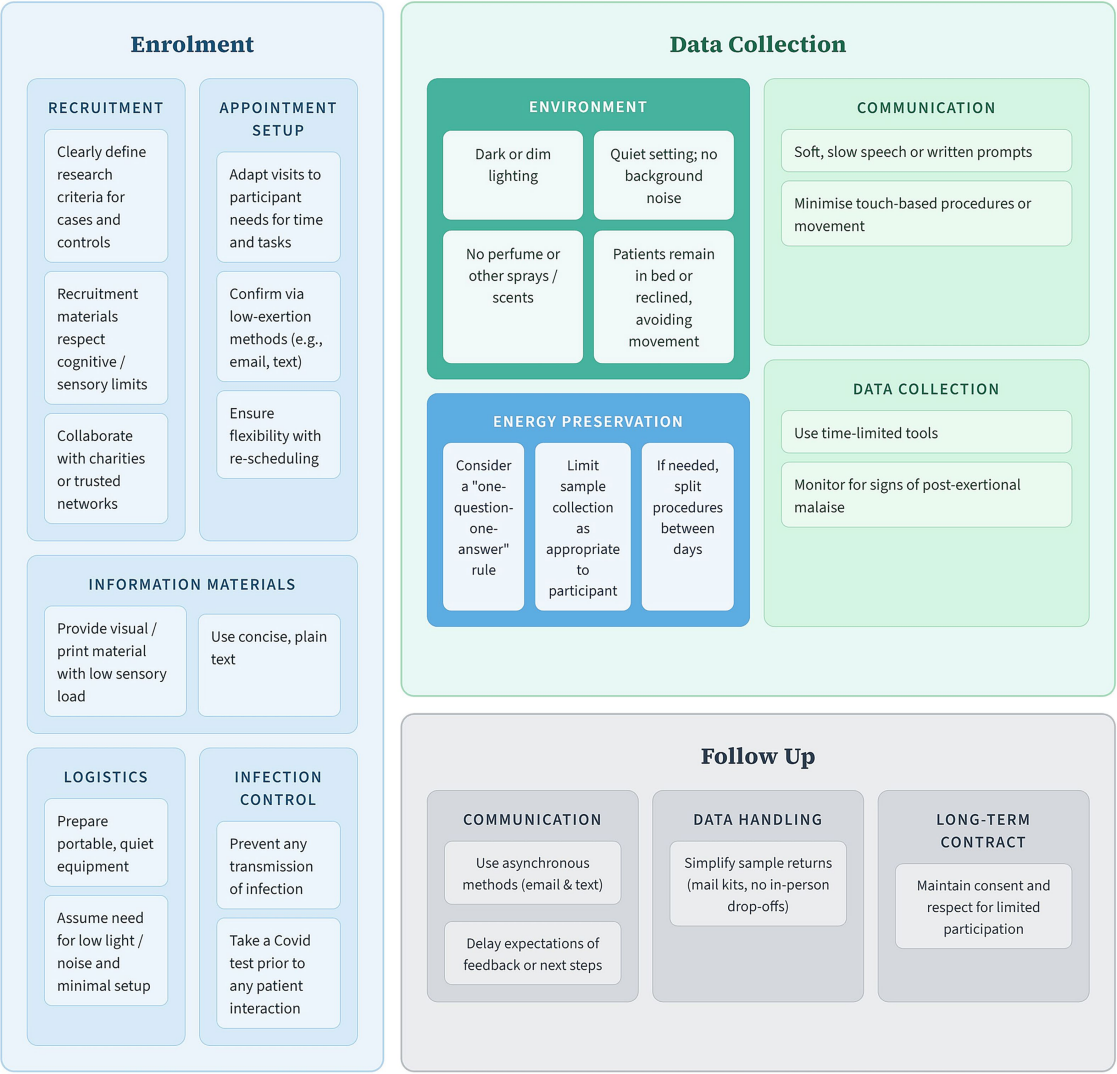
The CureME Model: framework for inclusive, home-based - and clinic-based ME/CFS research visits across severity levels. The CureME Model: a framework for inclusive ME/CFS research across the disease severity spectrum. The framework outlines key considerations across three stages of research participation—Enrolment, Data Collection, and Follow Up—to guide the planning and conduct of data and sample collection visits. It is applicable across all severity levels and adaptable to both home- and clinic-based research settings.

As a result, building and maintaining trust with the ME/CFS community is essential for successful research engagement. This occurs through meaningful patient and public involvement and engagement (PPIE), operating across multiple levels of participation, as illustrated in Table 1. The CureME Framework works at Levels 6–8, involving people with ME/CFS as co-leaders or co-producers facilitating reciprocal learning, shared governance, and adapted participation methods (Kingdon et al., 2020; Lacerda et al., 2018; Holtzman et al., 2019; Devereux-Cooke et al., 2022). This requires considerable time and resources to build collaborative relationships with people with ME/CFS and other stakeholders, including charities and clinical services. In the CureME Framework, we use iterative consultation and shared decision-making to foster trust and co-design protocols covering recruitment, selection of outcome measures and assessment procedures (Figure 1). As a result, we achieve elevated levels of participant satisfaction and retention, even among severely affected individuals (Kingdon et al., 2020; Lacerda et al., 2018). In contrast, research conducted at Levels 1–4 risks perpetuating exclusion, may fail to accommodate participants’ needs, and could erode community trust (Lacerda et al., 2018; Arnstein, 1969; Chu et al., 2018; Jason et al., 2020).

**Table 1:** Levels of patient and public involvement and engagement (PPIE) in ME/CFS research.

Level	Title	Description	Example in ME/CFS research	Key references
8	Co-production/shared leadership	Patients co-lead all phases including design, governance, and dissemination with shared decision-making and reciprocal learning.	PWME included via adaptations allowing full participation.	Lacerda et al. (2017) and World Health Organization (2016)
7	Patient-led initiatives	Patients propose research questions or goals; researchers provide technical/logistical support.	Patient networks identify research priorities and collaborate with researchers on grant applications (e.g., DecodeME, genetic study).	Devereux-Cooke et al. (2022)
6	Deep consultation	Patients provide structured, iterative feedback via advisory boards or pilot testing.	Remote patient advisory groups review and refine consent materials, symptom assessment, protocols and study communications.	Thoma et al. (2024)
5	Functional involvement	Patient input informs study design, but researchers retain final decision authority; input may be episodic rather than continuous.	Patient advisors contribute to ethics panels, acknowledging participant vulnerabilities, but may not be involved in final decisions.	Collin et al. (2016)
4	Tokenism	Patients are named on committees but have limited opportunity to provide substantive input or influence the decisions.	Patient listed on steering committee but excluded from protocol development or key decision-makings.	Lacerda et al. (2017) and Bastos et al. (2025)
3	Information dissemination	Information was shared one-way without opportunity for patient feedback or influence.	Researchers post updates online or distribute newsletters about completed studies without soliciting input.	Standard practice in many early studies
2	Outreach without feedback	Patients contacted solely for recruitment, with no ongoing engagement or input into study design or conduct.	Recruitment materials distributed without explanation of study aim, result sharing or mechanisms for feedback.	Nacul et al. (2020)
1	Complete exclusion	No patient input: protocols developed exclusively by researchers, often neglecting patient needs such as PEM triggers, cognitive demands and accessibility requirements.	Studies designed without accommodations for PEM or communication need; data collection methods incompatible with severe illness.	British Association of Clinicians in ME (BACME) (2023)

This table illustrates the spectrum of engagement approaches and their implications for study quality, participant trust, and inclusivity. Higher levels of engagement are associated with improved methodological rigor and better accommodation of participant needs. Levels adapted from Arnstein’s (1969) and INVOLVE’s framework for public involvement in research. Higher levels (Carruthers et al., 2011; Hartle et al., 2021; Stussman et al., 2020) are associated with improved study feasibility, participant retention, and scientific rigor in ME/CFS research.

While co-production and participatory approaches offer significant benefits, researchers should remain aware of potential limitations and implement strategies to minimize bias and unequal participation. PPIE contributors and steering group members should reflect the diversity of the wider ME/CFS population, including underrepresented groups such as ethnic minorities, individuals experiencing socio-economic disadvantage, people with severe ME/CFS, and men, who may be less represented due to the higher prevalence of ME/CFS in women. Additionally, discussions and decision-making processes should be carefully facilitated to promote equitable participation and prevent individuals with particularly strong views from disproportionately influencing outcomes.

### 2.2 Practical barriers to study participation

In this section, we detail practical strategies for inclusive recruitment, accessible data collection, and effective participant retention that exemplify Level 6–8 engagement (Table 1).

As noted above, people with ME/CFS need to carefully manage their activity and energy levels to avoid PEM. Therefore, research studies should be designed to minimize both physical exertion and cognitive load. However, risk assessments and study protocols need to explicitly address the risk that participation may trigger PEM, even with mitigation strategies in place.

Therefore, the CureME Framework recommends working with ME/CFS charities, clinicians, individuals with ME/CFS and their caregivers (Brett et al., 2014; Lacerda et al., 2018) to ensure these needs are recognized and accommodated. The model suggests progression from participatory focus group work to the establishment of a steering committee, which includes people with ME/CFS (Lacerda et al., 2010; Lacerda et al., 2017). This committee then collaboratively shapes recruitment strategies, outreach approaches, production of materials, and data collection procedures (Lacerda et al., 2018; Lacerda et al., 2010; Lacerda et al., 2017). Given participants’ fluctuating energy and the need to balance research involvement with daily life, study schedules should be flexible. This includes accommodating variations in timing of appointments and task duration incorporating rest periods allowing participants to pace themselves.

Fatigue can significantly impair information processing, attention span and ability to follow instructions (Wirth et al., 2021; Lee et al., 2024). So, simplified instructions with plain language, minimal extraneous detail, and ‘easy access’ formatting will minimize cognitive and sensory load, while reminders support memory function. Both should be included as standard. Even routine conversation can prove cognitively demanding. Therefore, communication must be clear, concise, and individualized to respect both cognitive limitations and participant autonomy. This will maintain clarity and study continuity while ensuring ethical and reliable data collection. Additionally, implementing ‘autosave’ functionality in electronic data collection instruments (e.g., online questionnaires) enables participants to complete assessments incrementally according to their individual pacing strategies (Samms and Ponting, 2025b; Lee et al., 2024; Deumer et al., 2021; Jain et al., 2017; Baxter, 2022; Boulazreg and Rokach, 2020).

Also, researchers should consider environmental factors when samples or data are being collected (see Figure 1), as some people with ME/CFS (particularly those who are severely affected) can be sensitive to visual, auditory, olfactory, and tactile stimuli. Therefore, environmental triggers such as bright lights, noisy and crowded spaces, and physical contact should be minimized, and fragrances avoided. As orthostatic intolerance is common in ME/CFS (Deumer et al., 2021), recumbent seating or a place where participants can lie down to rest will enable more people to participate. Taking these factors into consideration and given the additional burden of traveling to a research site, researchers should offer home visits when possible.

Further, immunological vulnerability and awareness of the potential risks of infection are important considerations. Consequently, researchers should avoid contact with participants if they are unwell, comply with any requests from participants, and take careful infection control measures. This includes wearing a mask at home visits, taking a SARS-CoV-2 test beforehand, stringent hand-washing, wearing a mask as appropriate, and removing shoes when entering a home for a visit. Other procedures that enable pacing to make home visits more feasible include advance distribution of sample collection kits and acceptance of self-reported anthropometric measurements. Home visit protocols should be systematically co-developed with relevant stakeholders during the grant application stage to ensure methodological rigor, feasibility, and participant safety.

Finally, a substantial proportion of potential participants are unable to work and may have limited income. Therefore, studies should provide full reimbursement for travel and other study-related expenses.

The flexibility and inclusivity offered in the CureME Framework improves recruitment and retention rates by ensuring the research processes are feasible and acceptable for participants and representative cohorts are recruited. However, the potential disadvantage of greater representativeness is sample heterogeneity, and greater flexibility can impact data quality. ME/CFS involves a vis heterogenous; in the variety of symptoms, range of disability, and multitude of biological abnormalities. Many studies have suggested phenotypes based on these factors (Collin et al., 2016; Bastos et al., 2025; Nacul et al., 2020), However none have been replicated, nor implemented. Further research to consolidate these pilot studies and establish effective phenotyping methods is urgently needed. In the meantime, researchers need to be as inclusive as possible, rigorously record their selection criteria and recruitment strategies, while acknowledging the limitations and excluded groups.

While the flexibility promoted by the CureME Framework enhances inclusivity, it may also compromise data standardization and quality. Researchers must carefully consider the trade-off between achieving a representative cohort and maintaining a sufficiently standardized dataset, ensuring that these methodological choices are transparently reported and justified.

### 2.3 Including people with severe ME/CFS

Approximately 25% of people with ME/CFS are severely affected, meaning they are largely housebound or bedbound. Individuals often experience profound cognitive and physical dysfunction and heightened sensitivity to sensory stimuli (Jain et al., 2017; Baxter, 2022). This level of disability often results in social isolation, and many have disengaged from, or lack access to healthcare providers (Boulazreg and Rokach, 2020; Hunt et al., 2024; Blease et al., 2017). This prevents their recruitment through clinical services (Hunt et al., 2022; Beentjes et al., 2024; Brett et al., 2014; Baxter, 2022). Consequently, people with severe ME/CFS are under-represented in most research, undermining the representativeness of study samples and limiting understanding of the disease’s full spectrum (O’Boyle et al., 2022; McDonald et al., 2022; Richman et al., 2019). Researchers need to be prepared to use all the strategies outlined above, particularly home visits and to negotiate how to meet participants’ needs. To reduce participant burden further, research systems should allow participants to assist by relatives, caregivers, or a proxy with support documented to maintain data integrity.

Having emphasized the importance and benefits of involving people with severe ME/CFS, researchers should be aware that some will be too ill and disabled to participate, regardless of the study design, or the accommodation offered. Careful discussion with potential participants, or their proxy, is essential to ensure they are aware of any risks and the measures to minimize those risks, to ensure they can provide fully informed consent, or decide against participation.

## 3 Conclusion

ME/CFS research cannot rely on conventional research approaches and processes, which are limited by diagnostic ambiguity, historical distrust and logistical barriers. We present the CureME Framework as a way to address these challenges. It involves meaningful involvement of people with ME/CFS, and a flexible approach using participatory recruitment models and adaptive study designs, resulting in greater cohort representativeness and more robust study outcomes.

## Data Availability

The original contributions presented in the study are included in the article/supplementary material, further inquiries can be directed to the corresponding author.

## References

[ref1] Arnstein S. R. (1969). A ladder of citizen participation. J. Am. Inst. Plann. 35, 216–224. doi: 10.1080/01944366908977225

[ref2] Arron H. E. Marsh B. D. Kell D. B. Khan M. A. Jaeger B. R. Pretorius E. (2024). Myalgic encephalomyelitis/chronic fatigue syndrome: the biology of a neglected disease. Front. Immunol. 15:1386607. doi: 10.3389/fimmu.2024.1386607, 38887284 PMC11180809

[ref3] Baker D. Hann M. (2001). General practitioner services in primary care groups in England: is there inequity between service availability and population need? Health Place 7, 67–74. doi: 10.1016/S1353-8292(00)00041-1, 11470220

[ref4] Bastos V. C. Greene K. A. Tabachnikova A. Bhattacharjee B. Sjögren P. Bertilson B. . (2025). Cerebrospinal fluid immune phenotyping reveals distinct immunotypes of myalgic encephalomyelitis/chronic fatigue syndrome. J. Immunol. 214, 1539–1551. doi: 10.1093/jimmun/vkaf087, 40373264 PMC12311384

[ref5] Baxter H. (2022). Ensuring the voice of the very severely affected Myalgic encephalomyelitis/chronic fatigue syndrome patient is heard in research-a research model. Healthcare (Basel) 10:1278. doi: 10.3390/healthcare10071278, 35885805 PMC9319152

[ref6] Bayliss K. Goodall M. Chisholm A. Fordham B. Chew-Graham C. Riste L. . (2014). Overcoming the barriers to the diagnosis and management of chronic fatigue syndrome/ME in primary care: a meta synthesis of qualitative studies. BMC Fam. Pract. 15:44. doi: 10.1186/1471-2296-15-44, 24606913 PMC3973969

[ref7] Beentjes S. V. Kaczmarczyk J. Cassar A. Samms G. L. Hejazi N. S. Khamseh A. . (2024). Replicated blood-based biomarkers for Myalgic encephalomyelitis not explicable by inactivity. EMBO Molecular Medicine. doi: 10.1101/2024.08.26.24312606 PMC12254397 40537675

[ref8] Blease C. Carel H. Geraghty K. (2017). Epistemic injustice in healthcare encounters: evidence from chronic fatigue syndrome. J. Med. Ethics 43, 549–557. doi: 10.1136/medethics-2016-103691, 27920164

[ref9] Boulazreg S. Rokach A. (2020). The lonely, isolating, and alienating implications of Myalgic encephalomyelitis/chronic fatigue syndrome. Healthcare (Basel) 8:413. doi: 10.3390/healthcare8040413, 33092097 PMC7711762

[ref10] Brett J. Staniszewska S. Mockford C. Herron-Marx S. Hughes J. Tysall C. . (2014). A systematic review of the impact of patient and public involvement on service users, researchers and communities. Patient 7, 387–395. doi: 10.1007/s40271-014-0065-0, 25034612

[ref11] British Association of Clinicians in ME (BACME) (2023). ME/CFS National Services Survey 2023. London, England: British Association of Clinicians in ME/CFS (BACME). Available online at: https://bacme.info/wp-content/uploads/2023/10/BACME-National-Services-Survey-Report-Oct23.pdf (Accessed June 19, 2026).

[ref12] Brurberg K. G. Fønhus M. S. Larun L. Flottorp S. Malterud K. (2014). Case definitions for chronic fatigue syndrome/myalgic encephalomyelitis (CFS/ME): a systematic review. BMJ Open 4:e003973. doi: 10.1136/bmjopen-2013-003973, 24508851 PMC3918975

[ref13] Carruthers B. M. van de Sande M. I. De Meirleir K. L. Klimas N. G. Broderick G. Mitchell T. . (2011). Myalgic encephalomyelitis: international consensus criteria. J. Intern. Med. 270, 327–338. doi: 10.1111/j.1365-2796.2011.02428.x, 21777306 PMC3427890

[ref14] Chu L. Valencia I. J. Garvert D. W. Montoya J. G. (2018). Deconstructing post-exertional malaise in myalgic encephalomyelitis/chronic fatigue syndrome: a patient-centered, cross-sectional survey. PLoS One 13:e0197811. doi: 10.1371/journal.pone.0197811, 29856774 PMC5983853

[ref15] Collin S. M. Nikolaus S. Heron J. Knoop H. White P. D. Crawley E. (2016). Chronic fatigue syndrome (CFS) symptom-based phenotypes in two clinical cohorts of adult patients in the UK and the Netherlands. J. Psychosom. Res. 81, 14–23. doi: 10.1016/j.jpsychores.2015.12.006, 26800634

[ref16] Committee on the Diagnostic Criteria for Myalgic Encephalomyelitis/Chronic Fatigue Syndrome and Board on the Health of Select Populations (2015). Beyond Myalgic Encephalomyelitis/Chronic Fatigue Syndrome: Redefining an Illness. Washington (DC): National Academies Press. 25695122

[ref17] Committee on the Diagnostic Criteria for Myalgic Encephalomyelitis/Chronic Fatigue Syndrome, Board on the Health of Select Populations, Institute of Medicine. Beyond Myalgic Encephalomyelitis/Chronic Fatigue Syndrome: Redefining an Illness. Washington (DC): The National Academies Press; (2015). Available online at: http://www.nap.edu/catalog.php?record_id=19012 (Accessed June 19, 2026) 25695122

[ref18] Conroy K. E. Islam M. F. Jason L. A. (2022). Evaluating case diagnostic criteria for myalgic encephalomyelitis/chronic fatigue syndrome (ME/CFS): toward an empirical case definition. Disabil. Rehabil. 45, 840–847. doi: 10.1080/09638288.2022.2043462, 35236205 PMC9437146

[ref19] Deumer U. S. Varesi A. Floris V. Savioli G. Mantovani E. López-Carrasco P. . (2021). Myalgic encephalomyelitis/chronic fatigue syndrome (ME/CFS): an overview. J. Clin. Med. 10:4786. doi: 10.3390/jcm10204786, 34682909 PMC8538807

[ref20] Devereux-Cooke A. Leary S. McGrath S. J. Northwood E. Redshaw A. Shepherd C. . (2022). DecodeME: community recruitment for a large genetics study of myalgic encephalomyelitis / chronic fatigue syndrome. BMC Neurol. 22:269. doi: 10.1186/s12883-022-02763-6, 35854226 PMC9294749

[ref21] Esmail L. Moore E. Rein A. (2015). Evaluating patient and stakeholder engagement in research: moving from theory to practice. J Comp Eff Res. 4, 133–145. doi: 10.2217/cer.14.79, 25825842

[ref22] Froehlich L. Hattesohl D. B. Cotler J. Jason L. A. Scheibenbogen C. Behrends U. (2021). Causal attributions and perceived stigma for myalgic encephalomyelitis/chronic fatigue syndrome. J. Health Psychol. 27, 2291–2304. doi: 10.1177/13591053211027631, 34240650 PMC9434257

[ref23] Fukuda K. Straus S. E. Hickie I. Sharpe M. C. Dobbins J. G. Komaroff A. (1994). The chronic fatigue syndrome: a comprehensive approach to its definition and study. Ann. Intern. Med. 121, 953–959. doi: 10.7326/0003-4819-121-12-199412150-00009, 7978722

[ref24] Goodman M. S. Ackermann N. Bowen D. J. panel D Thompson V. S. (2020). Reaching consensus on principles of stakeholder engagement in research. Prog. Community Health Partnersh. 14, 117–127. doi: 10.1353/cpr.2020.0014, 32280129 PMC7867997

[ref25] Hartle M. Bateman L. Vernon S. D. (2021). Dissecting the nature of post-exertional malaise. Fatigue 9, 33–44. doi: 10.1080/21641846.2021.1905415

[ref26] Holtzman C. S. Bhatia S. Cotler J. Jason L. A. (2019). Assessment of post-exertional malaise (PEM) in patients with Myalgic encephalomyelitis (ME) and chronic fatigue syndrome (CFS): a patient-driven survey. Diagnostics. 9:26. doi: 10.3390/diagnostics9010026, 30832336 PMC6468435

[ref27] Huang K. G C de Sá A. Thomas N. Phair R. D. Gooley P. R. Ascher D. B. . (2024). Discriminating Myalgic encephalomyelitis/chronic fatigue syndrome and comorbid conditions using metabolomics in UK biobank. Commun. Med. 4:248. doi: 10.1038/s43856-024-00669-7, 39592839 PMC11599898

[ref28] Hunt J. Blease C. Geraghty K. J. (2022). Long Covid at the crossroads: comparisons and lessons from the treatment of patients with myalgic encephalomyelitis/chronic fatigue syndrome (ME/CFS). J. Health Psychol. 27, 3106–3120. doi: 10.1177/13591053221084494, 35341334

[ref29] Hunt J. Runacres J. Herron D. Sheffield D. (2024). Exploring the experience of healthcare-related epistemic injustice among people with Myalgic encephalomyelitis/chronic fatigue syndrome. Qual. Rep. 53. doi: 10.46743/2160-3715/2024.6519

[ref30] Hvidberg M. F. Brinth L. S. Olesen A. V. Petersen K. D. Ehlers L. (2015). The health-related quality of life for patients with myalgic encephalomyelitis / chronic fatigue syndrome (ME/CFS). PLoS One 10:e0132421. doi: 10.1371/journal.pone.0132421, 26147503 PMC4492975

[ref31] Jain V. Arunkumar A. Kingdon C. Lacerda E. Nacul L. (2017). Prevalence of and risk factors for severe cognitive and sleep symptoms in ME/CFS and MS. BMC Neurol. 17:117. doi: 10.1186/s12883-017-0896-0, 28633629 PMC5477754

[ref32] Jason L. A. Benton M. C. Valentine L. Johnson A. Torres-Harding S. (2008). The economic impact of ME/CFS: individual and societal costs. Dyn. Med. 7:6. doi: 10.1186/1476-5918-7-6, 18397528 PMC2324078

[ref33] Jason L. A. Evans M. So S. Scott J. Brown A. (2015). Problems in defining post-exertional malaise. J. Prev. Interv. Community 43, 20–31. doi: 10.1080/10852352.2014.973239, 25584525 PMC4295644

[ref34] Jason L. A. Katz B. Z. Sunnquist M. Torres C. Cotler J. Bhatia S. (2020). The prevalence of pediatric Myalgic encephalomyelitis/chronic fatigue syndrome in a community-based sample. Child Youth Care Forum 49, 563–579. doi: 10.1007/s10566-019-09543-3, 34113066 PMC8186295

[ref35] Jones C. L. Younger J. (2025). Possible racial disparities in the diagnosis of Myalgic encephalomyelitis/chronic fatigue syndrome (ME/CFS). Int. J. Environ. Res. Public Health 22:280. doi: 10.3390/ijerph22020280, 40003505 PMC11854918

[ref36] Kindlon T. (2011). The PACE trial in chronic fatigue syndrome. Lancet 377:1833. doi: 10.1016/S0140-6736(11)60684-3, 21592560

[ref37] Kingdon C. C. Bowman E. W. Curran H. Nacul L. Lacerda E. M. (2018). Functional status and well-being in people with Myalgic encephalomyelitis/chronic fatigue syndrome compared with people with multiple sclerosis and healthy controls. Pharmacoecon Open. 2, 381–392. doi: 10.1007/S41669-018-0071-6, 29536371 PMC6249197

[ref38] Kingdon C. Giotas D. Nacul L. Lacerda E. (2020). Health care responsibility and compassion-visiting the housebound patient severely affected by ME/CFS. Healthcare. 8:197. doi: 10.3390/healthcare8030197, 32635535 PMC7551603

[ref39] Lacerda E. M. Bowman E. W. Cliff J. M. Kingdon C. C. King E. C. Lee J. S. S. . (2017). The UK ME/CFS biobank for biomedical research on Myalgic encephalomyelitis/chronic fatigue syndrome (ME/CFS) and multiple sclerosis. Open J. Bioresour. 4:4. doi: 10.5334/ojb.28, 28649428 PMC5482226

[ref40] Lacerda E. M. Kingdon C. C. Bowman E. W. Nacul L. (2018). Using a participatory approach to develop and implement the UK ME/CFS biobank. Fatigue. 6, 1–4. doi: 10.1080/21641846.2018.1396021, 29938127 PMC6005931

[ref41] Lacerda E. M. McDermott C. Kingdon C. C. Butterworth J. Cliff J. M. Nacul L. (2019). Hope, disappointment and perseverance: reflections of people with Myalgic encephalomyelitis/chronic fatigue syndrome (ME/CFS) and multiple sclerosis participating in biomedical research. A qualitative focus group study. Health Expect. 22, 373–384. doi: 10.1111/hex.12857, 30632248 PMC6543144

[ref42] Lacerda E. M. Mudie K. Kingdon C. C. Butterworth J. D. O’Boyle S. Nacul L. (2018). The UK ME/CFS biobank: a disease-specific biobank for advancing clinical research into Myalgic encephalomyelitis/chronic fatigue syndrome. Front. Neurol. 9, 1–7. doi: 10.3389/fneur.2018.01026, 30564186 PMC6288193

[ref43] Lacerda E. M. Nacul L. Pheby D. Shepherd C. Spencer P. (2010). Exploring the feasibility of establishing a disease-specific post-mortem tissue bank in the UK: a case study in ME/CFS. J. Clin. Pathol. 63, 1032–1034. doi: 10.1136/jcp.2010.082032, 20924033

[ref44] Lee J. S. Sato W. Son C. G. (2024). Brain-regional characteristics and neuroinflammation in ME/CFS patients from neuroimaging: a systematic review and meta-analysis. Autoimmun. Rev. 23:103484. doi: 10.1016/j.autrev.2023.103484, 38016575

[ref45] Lim E. J. Son C. G. (2020). Review of case definitions for myalgic encephalomyelitis/chronic fatigue syndrome (ME/CFS). J. Transl. Med. 18:289. doi: 10.1186/s12967-020-02455-0, 32727489 PMC7391812

[ref46] McDonald S. Tan S. X. Banu S. van Driel M. McGree J. M. Mitchell G. . (2022). Exploring symptom fluctuations and triggers in myalgic encephalomyelitis/chronic fatigue syndrome using novel patient-centred N-of-1 observational designs: a protocol for a feasibility and acceptability study. Patient 15, 197–206. doi: 10.1007/s40271-021-00540-0, 34368926

[ref47] Muirhead N. L. Vyas J. Ephgrave R. Singh R. Finlay A. Y. (2024). Myalgic encephalomyelitis/chronic fatigue syndrome: impact on quality of life (QoL) of persons with ME/CFS. Medicina (B Aires) 60:1215. doi: 10.3390/medicina60081215, 39202496 PMC11356561

[ref48] Nacul L. Kingdon C. C. Bowman E. W. Curran H. Lacerda E. M. L Kingdon N. C. C. . (2017). Differing case definitions point to the need for an accurate diagnosis of myalgic encephalomyelitis/chronic fatigue syndrome. Fatigue Biomed. Health Behav. 5, 1–4. doi: 10.1080/21641846.2017.1273863, 29250461 PMC5730342

[ref49] Nacul L. C. Lacerda E. M. Campion P. Pheby D. Drachler M. d. L. Leite J. C. . (2011). The functional status and well being of people with myalgic encephalomyelitis/chronic fatigue syndrome and their carers. BMC Public Health 11:402. doi: 10.1186/1471-2458-11-402, 21619607 PMC3123211

[ref50] Nacul L. Lacerda E. M. Kingdon C. C. Curran H. Bowman E. W. (2018). How have selection bias and disease misclassification undermined the validity of myalgic encephalomyelitis/chronic fatigue syndrome studies? J. Health Psychol. 24, 1765–1769. doi: 10.1177/1359105317695803, 28810428 PMC5581258

[ref51] Nacul L. C. Lacerda E. M. Pheby D. Campion P. Molokhia M. Fayyaz S. . (2011). Prevalence of myalgic encephalomyelitis/chronic fatigue syndrome (ME/CFS) in three regions of England: a repeated cross-sectional study in primary care. BMC Med. 9:91. doi: 10.1186/1741-7015-9-91, 21794183 PMC3170215

[ref52] Nacul L. O’Boyle S. Palla L. Nacul F. E. Mudie K. Kingdon C. C. . (2020). How Myalgic encephalomyelitis/chronic fatigue syndrome (ME/CFS) progresses: the natural history of ME/CFS. Front. Neurol. 11:826. doi: 10.3389/fneur.2020.00826, 32849252 PMC7431524

[ref53] National Guideline Centre (UK) (2020). Identifying and Diagnosing ME/CFS: Myalgic Encephalomyelitis (or Encephalopathy)/Chronic Fatigue Syndrome: Diagnosis and Management Evidence Review. London: National Institute for Health and Care Excellence (NICE). 35438857

[ref54] O’Boyle S. Nacul L. Nacul F. E. Mudie K. Kingdon C. C. Cliff J. M. . (2022). A natural history of disease framework for improving the prevention, management, and research on post-viral fatigue syndrome and other forms of Myalgic encephalomyelitis/chronic fatigue syndrome. Front Med (Lausanne) 8:8159. doi: 10.3389/fmed.2021.688159, 35155455 PMC8835111

[ref55] Pendergrast T. Brown A. Sunnquist M. Jantke R. Newton J. L. Strand E. B. . (2016). Housebound versus nonhousebound patients with myalgic encephalomyelitis and chronic fatigue syndrome. Chronic Illn. 12, 292–307. doi: 10.1177/1742395316644770, 27127189 PMC5464362

[ref56] Pheby D. F. H. Araja D. Berkis U. Brenna E. Cullinan J. de Korwin J. D. . (2020). A literature review of GP knowledge and understanding of ME/CFS: a report from the socioeconomic working group of the European network on ME/CFS (EUROMENE). Medicina (B Aires) 57:7. doi: 10.3390/medicina57010007, 33374291 PMC7823627

[ref57] Richman S. Morris M. C. Broderick G. Craddock T. J. A. Klimas N. G. Fletcher M. A. (2019). Pharmaceutical interventions in chronic fatigue syndrome: a literature-based commentary. Clin. Ther. 41, 798–805. doi: 10.1016/j.clinthera.2019.02.011, 30871727 PMC6543846

[ref58] Samms G. L. Ponting C. P. (2025a). Defining a high-quality Myalgic encephalomyelitis/chronic fatigue syndrome cohort in UK biobank. NIHR Open Res. 5:39. doi: 10.3310/nihropenres.13956.1, 40443420 PMC12120426

[ref59] Samms G. L. Ponting C. P. (2025b). Unequal access to diagnosis of myalgic encephalomyelitis in England. BMC Public Health 25:1417. doi: 10.1186/s12889-025-22603-9, 40259275 PMC12012970

[ref60] Stussman B. Williams A. Snow J. Gavin A. Scott R. Nath A. . (2020). Characterization of post-exertional malaise in patients with Myalgic encephalomyelitis/chronic fatigue syndrome. Front. Neurol. 11:1025. doi: 10.3389/fneur.2020.01025, 33071931 PMC7530890

[ref61] Thoma M. Froehlich L. Hattesohl D. B. R. Quante S. Jason L. A. Scheibenbogen C. (2024). Why the psychosomatic view on Myalgic encephalomyelitis/chronic fatigue syndrome is inconsistent with current evidence and harmful to patients. Medicina 60:83. doi: 10.3390/medicina60010083, 38256344 PMC10819994

[ref62] Wallerstein N. B. Duran B. (2006). Using community-based participatory research to address health disparities. Health Promot. Pract. 7, 312–323. doi: 10.1177/1524839906289376 16760238

[ref63] Wiborg J. F. van der Werf S. Prins J. B. Bleijenberg G. (2010). Being homebound with chronic fatigue syndrome: a multidimensional comparison with outpatients. Psychiatry Res. 177, 246–249. doi: 10.1016/j.psychres.2010.02.010, 20207012

[ref64] Wilshire C. E. Kindlon T. (2019). Response: Sharpe, goldsmith and Chalder fail to restore confidence in the PACE trial findings. BMC Psychol. 7:19. doi: 10.1186/S40359-019-0296-X, 30914065 PMC6434781

[ref65] Wirth K. J. Scheibenbogen C. Paul F. (2021). An attempt to explain the neurological symptoms of Myalgic encephalomyelitis/chronic fatigue syndrome. J. Transl. Med. 19:471. doi: 10.1186/s12967-021-03143-3, 34809664 PMC8607226

[ref66] World Health Organization. International Statistical Classification of Diseases and Related Health Problems. 10th Revision, 5th ed. Vol. 3. Geneva: World Health Organization; (2016). Available online at: https://icd.who.int/browse10/2016/en (Accessed June 19, 2026)

